# Academic Well-Being, Mathematics Performance, and Educational Aspirations in Lower Secondary Education: Changes Within a School Year

**DOI:** 10.3389/fpsyg.2018.00297

**Published:** 2018-03-13

**Authors:** Anna Widlund, Heta Tuominen, Johan Korhonen

**Affiliations:** ^1^Faculty of Education and Welfare Studies, Åbo Akademi University, Vasa, Finland; ^2^Faculty of Educational Sciences, University of Helsinki, Helsinki, Finland

**Keywords:** academic well-being, school burnout, schoolwork engagement, self-concept, performance, mathematics, educational aspirations, person-centered approach

## Abstract

It has been suggested that both performance and academic well-being play a role in adolescent students’ educational attainment and school dropout. In this study, we therefore examined, first, what kinds of academic well-being (i.e., school burnout, schoolwork engagement, and mathematics self-concept) and mathematics performance profiles can be identified among lower secondary school students (*N*_grade 7_ = 583, *N*_grade 9_ = 497); second, how stable these profiles are across one school year during the seventh and ninth grades; and, third, how students with different academic well-being and mathematics performance profiles differ with respect to their educational aspirations. By means of latent profile analyses, three groups of students in seventh grade: thriving (34%), average (51%), and negative academic well-being (15%) and four groups of students in ninth grade: thriving (25%), average (50%), negative academic well-being (18%), and low-performing (7%) with distinct well-being and mathematics performance profiles were identified. Configural frequency analyses revealed that the profiles were relatively stable across one school year; 60% of the students displayed identical profiles over time. The thriving students reported the highest educational aspirations compared to the other groups. In addition, the low-performing students in the ninth grade had the lowest educational aspirations just before the transition to upper secondary school. Practical implications as well as directions for future research are discussed.

## Introduction

By utilizing a person-centered approach, the aim of this study was to investigate lower secondary school students’ academic well-being and mathematics performance profiles, the stability of the profiles during one school year, and their relations to educational aspirations. It has been demonstrated that performance plays a significant role in shaping students’ educational experiences, aspirations, and paths but, also, that academic well-being matters for various educational outcomes. More specifically, low academic well-being has been linked with, for example, low academic achievement, unfavorable motivational tendencies, learning difficulties, lower educational aspirations, educational delays, and dropout (e.g., Salmela-Aro et al., 2009b; Vasalampi et al., 2009; Tuominen-Soini et al., 2012; Bask and Salmela-Aro, 2013; Tuominen-Soini and Salmela-Aro, 2014; Korhonen et al., 2016; Fiorilli et al., 2017). Many of the existing studies on well-being and educational outcomes have used a variable-centered approach and, thus, focused on the whole sample averages. However, a person-centered focus is useful, whenever it is assumed that the data include heterogeneous groups of individuals. For instance, Korhonen et al. (2014) proved the person-centered approach to be advantageous for investigating the relations between academic well-being, performance (in mathematics and reading), and school dropout. Interestingly, not only students with low performance but also students with poor academic well-being were more prone to school dropout than students with high or average performance. It seems therefore reasonable to also investigate how the individual differences in students’ academic well-being and performance might jointly contribute to educational aspirations. As lower secondary school students are facing the transition to upper secondary school along with the first possibility to make a decision about their own education, it seems highly relevant to explore these relations during lower secondary years.

Moreover, there is evidence of an overall decline in academic achievement, motivation, and well-being during adolescence (e.g., Roeser et al., 1999; Watt, 2004; Eccles and Roeser, 2009) but, still, surprisingly few studies have investigated the developmental dynamics of academic well-being and performance during lower secondary education, especially by means of a person-centered approach. The person-centered focus is useful also with longitudinal data to represent heterogeneity in developmental trajectories. The existing studies using this kind of an analytical approach have, in fact, uncovered that not all students experience these negative shifts (Tuominen-Soini et al., 2011, 2012; Tuominen-Soini and Salmela-Aro, 2014). Accordingly, the aim of the present study was, also, to complement prior research by examining the stability of and changes in students’ academic well-being and mathematics performance profiles within one school year during lower secondary school, using two cohorts of students (i.e., seventh- and ninth-graders).

### Academic Well-Being

#### Conceptualization of Academic Well-Being

Since well-being is an important indicator for various educational outcomes (Tuominen-Soini et al., 2012; Fiorilli et al., 2017) and, given the centrality of school in the lives of adolescents (Eccles and Roeser, 2009), it is reasonable to define well-being in relation to the educational context (i.e., academic well-being). There is no consensus on the definition of students’ academic well-being, but it is often described as a multidimensional construct, comprising several sub-dimensions. In prior studies, academic well-being has been conceptualized as being comprised of, for example, academic self-concept, perceived learning difficulties, and school burnout (Korhonen et al., 2014), school burnout, schoolwork engagement, school value, and satisfaction with educational choice (Tuominen-Soini et al., 2012), and school burnout and engagement (Fiorilli et al., 2017). Therefore, in line with previous research and as recommended by Huppert and So (2013), we conceptualized well-being as a multidimensional construct covering both negative (e.g., burnout) and positive (e.g., engagement) aspects of academic well-being in our investigation. In addition, since specifically students’ math-related self-beliefs about their competencies have been found to predict educational and occupational aspirations and choices (Parker et al., 2013), we also defined and assessed well-being within a specific domain, in this case, mathematics. Consequently, school burnout, schoolwork engagement, and mathematics self-concept were chosen as indicators of academic well-being in the present study.

School burnout mirrors that of professional burnout (Maslach et al., 2001) and can be described as a psychological syndrome caused by long-term exposure to school-related stress and pressure to achieve (Schaufeli et al., 2002; Di Chiacchio et al., 2016). Salmela-Aro et al. (2009a) designed and validated the School Burnout Inventory (SBI-9), which measures three dimensions of school burnout; exhaustion due to school demands, cynical and detached attitudes toward one’s school, and feelings of inadequacy as a student. This instrument has been applied to several academic populations, with the same three-factor structure being confirmed in Finland (Salmela-Aro et al., 2009a), Spain (Boada-Grau et al., 2015), France (Faye-Dumanget et al., 2017), and Italy (Fiorilli et al., 2014). These three dimensions are closely related (Toppinen-Tanner et al., 2005), but differently associated with various school-related outcomes (e.g., achievement: Salmela-Aro and Upadyaya, 2014; motivation: Tuominen-Soini et al., 2008, 2012) and are therefore examined as distinct constructs in the present study.

Schoolwork engagement, also suggested to be a central indicator for well-being in school (Tuominen-Soini et al., 2012; Fiorilli et al., 2017), is defined as a positive, fulfilling, study-related feeling composed by three dimensions: vigor, dedication, and absorption (Salmela-Aro and Upadyaya, 2012). Vigor is characterized by high levels of energy while studying, interest and willingness to invest in schoolwork as well as having effective strategies for coping with difficulties. Being dedicated indicates being enthusiastic and having positive attitudes toward the learning processes and outcomes, while absorption refers to being fully concentrated and involved in one’s studies, finding it difficult to detach oneself from schoolwork.

Lastly, since students’ self-beliefs are commonly used indicators of adolescent well-being (e.g., self-esteem and competence, Huppert and So, 2013; self-esteem, Tuominen-Soini et al., 2008; Parhiala et al., 2018; academic self-concept, Korhonen et al., 2014), mathematics self-concept was chosen as an indicator of academic well-being as well. Academically related self-concept is described as the mental representation of one’s competencies in academic domains (Marsh and Craven, 1997).

The dimensions of school engagement and school burnout have been found to be negatively associated (Schaufeli et al., 2002; Salmela-Aro et al., 2009b; Cadime et al., 2016). They are, however, not two opposite ends of the spectrum, but rather, independent constructs (Schaufeli and Bakker, 2004). Despite the negative correlation, studies investigating engagement and burnout among adolescents using person-centered approaches have found students with high engagement and low burnout, students with low engagement and high burnout, and students who are simultaneously highly engaged in school and exhausted due to school demands (Tuominen-Soini and Salmela-Aro, 2014; Salmela-Aro et al., 2016). Similarly, Parhiala et al. (2018) recently investigated profiles of motivation and well-being among adolescent students by using a person-centered approach, and found a group of students with rather negative well-being (i.e., school burnout, internalizing and externalizing problems, low self-esteem), who were still highly motivated in school. These findings also resemble those found by Daniels et al. (2008) and Tuominen-Soini et al. (2008, 2012), indicating that high-achieving, committed, and motivated students who value schoolwork might, at the same time, be receptive to emotional distress and exhaustion. Regarding academic self-concept, it has been found to be positively related to engagement (Guo et al., 2016), lower school burnout (Korhonen et al., 2014) and, also, attitudes toward school (Green et al., 2012).

#### Academic Well-Being and Performance

As for the relation between well-being and academic performance, it has been found that the dimensions of school burnout are associated with academic performance in various ways. Salmela-Aro et al. (2009b) found that the lower the academic achievement and the lower the school engagement, the more cynicism toward the meaning of school and sense of inadequacy at school the adolescents experienced. Then again, exhaustion has been found to occur among high-achieving and motivated students as well (Tuominen-Soini et al., 2008, 2012). Schoolwork engagement has been found to have a direct, positive effect on academic achievement and can be seen as a key resource for students as they face different school-related demands. If there is an imbalance between resources and demands, where the perceived demands exceed the personal resources, it may lead to an increase in student burnout (Salmela-Aro and Upadyaya, 2014). Further, the positive link between academic self-concept and academic achievement is also well documented (Valentine et al., 2004; Möller et al., 2011), especially concerning domain-specific self-concept and achievement (Denissen et al., 2007; Guo et al., 2015b).

#### Stability and Change

Researchers generally regard adolescence as a period characterized by considerable biological, social, psychological, and developmental change. However, studies that have investigated school burnout throughout adolescence have found that the overall mean level of school burnout is quite stable over time, although some students seem to experience an increase in burnout during educational transitions (Salmela-Aro and Upadyaya, 2014). Also, as students grow older, they seem to become progressively more at risk of burnout (Lee et al., 2013; Fiorilli et al., 2017), possibly due to increased school demands. Engagement, on the other hand, has been found to decrease, possibly because of changes in the school environment when students transition from elementary to secondary school (Wang and Eccles, 2012). These declines could reflect an increasing misfit between students’ state of development related to adolescence and the opportunities provided in the secondary school environments (Eccles et al., 1993). Similarly, although academic self-concept has been found to become increasingly stable during adolescence, educational transitions seem to have a detrimental effect on students academic self-concept as well (Cole et al., 2001).

#### Gender Differences

Regarding well-being and gender differences, the overall trend seems to be that girls are more likely to report higher levels of school burnout than boys (Salmela-Aro et al., 2008; Salmela-Aro and Tynkkynen, 2012). However, although girls seem to be more receptive to school burnout, they are, at the same time, typically more engaged in schoolwork than boys (Salmela-Aro and Upadyaya, 2012) and attribute greater importance to academic achievement (Murberg and Bru, 2004). These results also concur with the findings by Parhiala et al. (2018), as they found that girls were overrepresented in groups characterized by rather high motivation but negative well-being.

When investigating gender differences in the domain of mathematics, a fairly consistent finding is that boys perform a little better in mathematics and have higher mathematics self-concept than girls (Nagy et al., 2006; Watt et al., 2012). The gender difference in mathematics in favor of boys seems to be the largest among the higher performing students and absent among the lower performing students (Stoet and Geary, 2013). Interestingly, gender differences in mathematics self-concept have also been found when there is no difference in the students’ mathematics performance (Pajares, 2005; Watt, 2006). These differences have often been explained in terms of gender stereotypes, that is, mathematics is commonly considered a “male” field. For example, Song et al. (2016) found that stereotypes about mathematics had both a direct and an indirect negative effect on girls’ mathematics performance.

### Educational Aspirations

One of the most significant predictors of actual educational and career attainment is educational aspirations (Mau and Bikos, 2000; Garg et al., 2007). There is no clear definition of or unified measurement to assess educational aspirations, but they are commonly described as students’ goals and plans within an academic setting (Trebbels, 2015), which can be divided into realistic and idealistic educational aspirations. Idealistic aspirations refer to the students’ desired attainment level, while realistic aspirations are described as the students’ actual perceived likelihood of success as well as more pragmatic expectations of completing the aspired level of education (Rojewski, 2005). However, although previous research have included both realistic and idealistic alternatives in the operationalisation of educational aspirations, no clear distinction between them have been made (Chow et al., 2012; Guo et al., 2015a). Therefore, we combine a realistic and an idealistic component to represent overall educational aspirations in the present study.

#### Educational Aspirations and Well-Being

Educational aspirations influence adolescents’ overall well-being later in life (Ashby and Schoon, 2012) and have previously mostly been predicted by socioeconomic background (Garg et al., 2007), performance-related indicators (e.g., grades), and different motivational beliefs (e.g., self-concept and interest; Nagy et al., 2006; Korhonen et al., 2016). However, investigations using students’ psychological well-being as a predictor of educational aspirations are scarce (for an exception, see Korhonen et al., 2016), even though well-being has been linked with several other important educational outcomes (e.g., achievement: Kiuru et al., 2008; motivational tendencies: Tuominen-Soini et al., 2012). Korhonen et al. (2014) investigated students’ academic performance and well-being profiles by using a person-centered approach, and found that students with low performance (in mathematics and reading) as well as students with poor academic well-being were more prone to school dropout than students with high or average performance. Therefore, one could expect academic well-being to have an effect on educational aspirations as well.

In a recent study by Korhonen et al. (2016), school burnout was found to have a negative indirect effect on educational aspirations through interest, but also, interestingly, that higher levels of school burnout were directly related to higher educational aspirations for girls. Since girls have been found to be overrepresented in groups of students with high levels of both engagement and school burnout (Tuominen-Soini and Salmela-Aro, 2014), it is possible that ambitious and success-oriented girls, despite feeling exhausted, still hold high educational goals (Tuominen-Soini et al., 2008; Tuominen-Soini and Salmela-Aro, 2014).

Researchers have found positive relations between students’ schoolwork engagement and educational and career aspirations (Kenny et al., 2006; Wang and Eccles, 2012). Hill and Wang (2015) revealed that this relation is reciprocal. Eccles (2007) suggested that students are more likely to engage in school if they understand the importance of engagement and achievement for their future. In contrast, it might be that students’ engagement in school also has the potential to shape goals and aspirations, as observing one’s own experiences at school provides the information needed to set up and meet educational goals (Bandura, 1991). Further, studies have found that students who are engaged with their schoolwork not only aspire for higher educational goals, but also complete higher education studies (Li and Lerner, 2011; Wang and Eccles, 2013).

Academic self-concept has consistently been found to be an important predictor of educational and career choices (Nagengast and Marsh, 2012; Parker et al., 2012) and aspirations (Korhonen et al., 2016), even when controlling for achievement (Parker et al., 2013). In a study by Guo et al. (2015a), academic self-concept was not only a key predictor of educational aspirations, but also a stronger predictor of long-term occupational aspirations and educational attainment than IQ and task values. Similarly, positive associations have been found between domain-specific mathematics self-concept and educational aspirations (Parker et al., 2013).

#### Educational Aspirations and Mathematics Performance

Previous research on mathematics performance and aspirations has focused primarily on the type of aspiration (i.e., the specific kind of occupation), particularly on the connection between mathematics performance and career choices in the science, technology, engineering, and mathematics (STEM) field. Overall, mathematics performance has been regarded as a critical filter, limiting later educational and occupational aspirations, and was therefore included in the present study. Shapka et al. (2006), for example, investigated the connections between early mathematics performance, gender, and career aspirations in ninth grade students and found, that students with lower grades in math had lower career aspirations than average- and high-performing students, even when controlling for overall academic achievement. Furthermore, mathematics performance has been found to predict educational aspirations even when controlling for relevant motivational and well-being constructs like interest, academic self-concept, and school burnout (Parker et al., 2014; Guo et al., 2015a,b; Korhonen et al., 2016).

### The Present Study

Given the importance of academic well-being for various educational outcomes (e.g., achievement: Fiorilli et al., 2017; motivation: Tuominen-Soini et al., 2012; educational aspirations: Korhonen et al., 2016), and considering that academic well-being fluctuates during adolescence, surprisingly few studies have investigated the development of academic well-being during lower secondary education. In fact, to our knowledge, none have examined within-year changes in academic well-being (i.e., school burnout, schoolwork engagement, and mathematics self-concept) and mathematics performance and, further, investigated the relation to educational aspirations over the course of lower secondary school. Also, many of the existing studies investigating the relation between well-being and performance, have used self-reports of students’ grades or grade point average as a measure of academic achievement, whereas we addressed this limitation of prior research by using a standardized mathematics test to assess students’ performance. This study advances the current knowledge base by investigating the stability of and change in students’ academic well-being and mathematics performance profiles by implementing a person-centered approach in two cohorts of lower secondary students and relating these profiles to educational aspirations. The following research questions were addressed:

(1) What kinds of academic well-being and mathematics performance profiles can be found among lower secondary school students (in seventh and ninth grade)?

Based on prior work (e.g., Korhonen et al., 2014), we expected to find certain well-being and performance profiles. Based on assumed linear relations between performance and well-being measures, three distinct groups of students were expected to emerge: students with high performance and positive academic well-being, students with average performance and moderate well-being, and students with low performance and rather negative well-being (H1). Further, since studies implementing a person-centered approach have found groups of students that are both highly engaged in their schoolwork but also perceive high levels of exhaustion (Daniels et al., 2008; Tuominen-Soini et al., 2008) and, in addition, students who show rather low levels of motivation in school but are still doing well in terms of well-being (Tuominen-Soini et al., 2008; Parhiala et al., 2018), we assumed that we might also find some sort of mixed profiles with non-linear relations between performance and academic well-being measures (H2). These apparent non-linear relationships between burnout, engagement, achievement, and aspirations might go unnoticed with traditional variable-centered methods, thus warranting a person-centered approach.

(2) How are girls and boys distributed within the profiles?

Based on prior work (Salmela-Aro et al., 2008; Salmela-Aro and Tynkkynen, 2012; Parhiala et al., 2018), we hypothesized that girls will be overrepresented in groups of students that are engaged in their schoolwork but also perceive higher levels of school burnout (H3). Also, since gender difference in mathematics among high-achieving students have been found in favor of boys (Stoet and Geary, 2013), we expected there to be more boys in the high-performing group (H4).

(3) How stable are these profiles during one school year?

As previous studies have revealed relative stability when investigating various patterns of well-being variables and performance (e.g., Roeser and Peck, 2003; Tuominen-Soini and Salmela-Aro, 2014), we expected to find some extent of stability in the profiles. In other words, that many students would stay in the same group across the school year (H5). Also, we assumed that more extreme changes in the group memberships over time would be rather rare, such as changes between the high performance and high well-being group and the low performance and low well-being group (H6), as well as changes between the high performance and high well-being group and the potential mixed performance and well-being group (H7). Further, students in the ninth grade are standing before an important decision of choosing an upper secondary education (i.e., academic or vocational track), while the seventh-graders, recently transitioned from elementary to lower secondary school, need to adjust to the new educational environment while simultaneously experiencing the turmoil of puberty. Therefore, and on the basis of previous results showing that transitional periods have an impact on students’ well-being (Wang and Eccles, 2012; Salmela-Aro and Upadyaya, 2014), we also expected some natural fluctuation to occur in the students’ well-being and performance profiles. However, as no previous study has investigated stability and change in academic well-being and performance profiles among adolescent students, no specific hypothesis was formulated for this assumption.

(4) How do students with different academic well-being and mathematics performance profiles differ with respect to their educational aspirations?

Since previous studies have shown that educational aspirations are connected to the level of both performance and well-being (Shapka et al., 2006; Korhonen et al., 2016), we hypothesized that students with the highest aspirations would come from the high-performing group (H8), while students with low performance and those with negative academic well-being would have the lowest aspirations (H9).

## Materials and Methods

### Context

Comprehensive schooling in Finland is comprised of primary school (grades 1–6) and lower secondary school (grades 7–9). The students attend comprehensive school for 9 years until 16 years of age. At the end of grade 9, students choose between either general upper secondary school (academic track) or vocational upper secondary school (vocational track), which both last 3 or 4 years. There is also a possibility to obtain double qualification, which implies attending courses in both general and vocational upper secondary schools. After completing either general or vocational upper secondary education, students are eligible to move into higher education, which has a dual structure in Finland; higher education is provided by universities and polytechnics, also known as universities of applied sciences (Finnish National Agency for Education, 2017). Universities emphasize scientific research and instruction, whereas universities of applied sciences adopt a more practical approach.

### Participants and Procedures

A total of 583 students in seventh grade (293 girls and 290 boys, mean age = 13.29 years, *SD* = 0.35) and 497 students in ninth grade (261 girls and 236 boys, mean age = 15.23 years, *SD* = 0.31) participated in the present study during one school year, 2016–2017. Participation in the study was voluntary, informed consent forms were collected from the students’ parents, and the participants were assured of the confidentiality of their responses. The students came from five lower secondary schools in different Swedish-speaking areas of Finland. Swedish is the second official language in Finland, where 5.3% of the population is Swedish speaking (*N* ≈ 290,000; Statistics Finland, 2016). Of the participating students, 54% had Swedish as their spoken language at home, while 28% spoke both Swedish and Finnish, 3% spoke Finnish and 4% spoke another language. The remaining participants did not report their home language. The students were followed up again at the end of the school year. The students completed self-report questionnaires on academic well-being and educational aspirations during one 45-min class session and a mathematics test during another 45-min class session.

### Measures

#### Mathematics Performance

The students’ mathematical skills were assessed with a standardized online assessment test (KTLT; Räsänen et al., 2013). The test development consisted of two steps. First, the candidate items with known difficulty level were selected from mathematical tasks originally used in the national assessments. Second, the item bank of 130 items were selected to be used in the test based on a sample of Finnish speaking students (*N* = 1157). All subjects solved subsamples of 40 items (with 10 anchor items in each test). The Cronbach alpha reliability was 0.89 (Räsänen and Leino, 2005). In this study the Finnish-Swedish translation of the battery was used. In this version the norms (IRT-values) were calculated from a sample of Finnish-Swedish speaking students (*N* = 1140) from grade levels 7–9 representing a national sample at that age.

The online assessment has three steps. First the student is asked to evaluate a difficulty of a single calculation task (easy, average, difficult). Based on the student’s answer, the system randomly selects the first item from a pool of items from easy, average, or hard items defined by their IRT delta value (difficulty parameter). In the second step, the system gives additional four items randomly from the pool of the items. In the third step, the system starts to recalculate a theta value (the estimated level of skill in the logit scale) after each given solution and selects the most informative item to be presented based on the delta and beta values (difficulty and discrimination parameters) from the remaining pool of items. The termination rule of the third step is that the theta value changes less than 2% from the current skill estimate after presenting a new item or that the subject has reached the maximum number of items (20) to be presented. To help the interpretation of the results the system transforms the student’s theta value automatically into a more familiar scale for educational practitioners using the test with a mean of 100 points (*SD* = 15). Likewise, the results in this study are presented using these transformed values.

#### Mathematics Self-Concept

Mathematics self-concept was measured with three items from Marsh’s (1992) Self Description Questionnaire I (SDQ I) scale (also see Arens and Hasselhorn, 2015). The items (e.g., *I learn things quickly in mathematics*) were assessed by a five-point Likert-type scale ranging from 1 (*completely false*) to 5 (*completely true*). The items were back-translated from English to Swedish.

#### Schoolwork Engagement

Schoolwork engagement was measured by the Schoolwork Engagement Inventory (EDA; Salmela-Aro and Upadyaya, 2012). The inventory consists of nine items measuring energy (e.g., *When I study I feel that I am bursting with energy*), dedication (e.g., *I am enthusiastic about my studies*), and absorption (e.g., *Time flies when I am studying*) in relation to schoolwork in general. The items were assessed through a seven-point Likert-type scale ranging from 0 (*never*) to 6 (*every day*). Both one- and three-factor solutions of schoolwork engagement are applicable when using the inventory (Salmela-Aro and Upadyaya, 2012). Accordingly, a composite score was computed from all items to indicate overall schoolwork engagement in the present study. The Swedish version of the inventory was obtained from the PISA2015 questionnaire (OECD, 2016).

#### School Burnout

School burnout was assessed by the nine-item School Burnout Inventory (SBI; Salmela-Aro et al., 2009a). The SBI scale is divided into three subscales: four items measuring emotional exhaustion (e.g., *I feel overwhelmed by my schoolwork*), three items measuring cynicism toward the meaning of school (e.g., *I feel that I am losing interest in my schoolwork*), and two items measuring the sense of inadequacy at school (e.g., *I often have feelings of inadequacy in my schoolwork*). This three-factor structure has been confirmed in several previous studies (see Salmela-Aro et al., 2009a; Fiorilli et al., 2014; Tuominen-Soini and Salmela-Aro, 2014). All items were assessed using a six-point Likert-type scale ranging from 1 (*completely disagree*) to 6 (*completely agree*). The Swedish version of the inventory was obtained from the PISA2015 questionnaire (OECD, 2016).

#### Educational Aspirations

We measured students’ idealistic and realistic educational aspirations according to two statements representing overall educational aspirations: *Highest academic degree I want to achieve* and *Highest academic degree I will probably achieve*, assessed using a 4-point ordinal scale (1 = *comprehensive education*, 2 = *vocational upper secondary education*, 3 = *polytechnic education*, and 4 = *university education*). These two items were combined to a composite score representing students educational aspirations.

Reliability coefficients and correlations between all the measures are reported in **Table 1**.

**Table 1:** Correlations, descriptive statistics and internal consistencies for all measures at Time 1 and Time 2.

		1	2	3	4	5	6	7	Grade 7 *M* (*SD*)	Grade 9 *M* (*SD*)
1	Mathematics performance	1/1	0.50^∗^/0.57^∗^	0.18^∗^/0.24^∗^	-0.11^∗^/-0.23^∗^	-0.20^∗^/-0.31^∗^	-0.17^∗^/-0.22^∗^	0.32^∗^/0.33^∗^	100.3/102.5 (12.8/15.5)	108.5/110.2 (15.0/16.0)
2	Mathematics self-concept	0.52^∗^/0.57^∗^	1/1	0.35^∗^/0.45^∗^	-0.37^∗^/-0.40^∗^	-0.35^∗^/-0.45^∗^	-0.35^∗^/-0.48^∗^	0.23^∗^/0.35^∗^	3.7/3.5 (0.85/0.97)	3.5/3.5 (0.95/0.92)
3	Engagement	0.22^∗^/0.25^∗^	0.31^∗^/0.34^∗^	1/1	-0.28^∗^/-0.22^∗^	-0.55^∗^/-0.56^∗^	-0.37^∗^/-0.37^∗^	0.30^∗^/0.32^∗^	4.3/4.1 (1.5/1.5)	4.1/4.0 (1.4/1.5)
4	Exhaustion	-0.09/-0.18^∗^	-0.26^∗^/-0.34^∗^	-0.13^∗^/-0.22^∗^	1/1	0.50^∗^/0.57^∗^	0.68^∗^/0.70^∗^	-0.09^∗^/-0.03	2.7/2.8 (1.1/1.2)	2.9/2.8 (1.2/1.2)
5	Cynicism	-0.11^∗^/-0.22^∗^	-0.23^∗^/-0.32^∗^	-0.43^∗^/-0.48^∗^	0.58^∗^/0.59^∗^	1/1	0.62^∗^/0.65^∗^	-0.28^∗^/-0.27^∗^	2.5/2.6 (1.1/1.2)	2.6/2.6 (1.1/1.2)
6	Inadequacy	-0.17^∗^/-0.21^∗^	-0.30^∗^/-0.39^∗^	-0.26^∗^/-0.34^∗^	0.69^∗^/0.71^∗^	0.62^∗^/0.65^∗^	1/1	-0.19^∗^/-0.12^∗^	2.8/2.8 (1.1/1.3)	3.1/3.0 (2.2/1.3)
7	Educational aspirations	0.32^∗^/0.35^∗^	0.27^∗^/0.31^∗^	0.38^∗^/0.32^∗^	0.05/-0.07	-0.20^∗^/-22^∗^	-0.14^∗^/-0.17^∗^	1/1	3.1/3.0 (0.83/0.80)	3.2/3.3 (0.77/0.72)
Cronbach’s alpha		-	0.92/0.93	0.94/95	0.80/83	0.78/81	0.60/65	–	–	–

^∗^*p < 0.05. Correlation coefficients above the diagonal refer to seventh grade students and under the diagonal to ninth grade students. Correlation coefficients for Time 1 and Time 2 are separated by slash*.

### Procedure

This study is part of the ongoing *Ungdomars Välbefinnande och Kunskap i Framtidens Samhälle* [Students’ well-being and learning in the future society] longitudinal study, following the students over a period of 4 years. The main aim of the project is to investigate the relations between well-being, achievement, and educational outcomes among adolescent students. A pilot study with 50 students was first conducted to assess the measurements. The measurements worked well in terms of length and clarity, and no changes were made based on the pilot study. Trained research assistants performed the first and the second data collection waves. Measurements were conducted with groups of students in their own schools, in intact classrooms during teacher-selected lessons.

### Data Analyses

We started the analyses by examining the patterns of missing data. The proportion of missing data in the variables at both time points ranged from 8.2 to 13.5%. The missing data were handled by imputing missing values with the expectation-maximization (EM) algorithm (Dempster et al., 1977). Second, we analyzed the structural stability of each well-being measure through longitudinal confirmatory factor analysis (LCFA). Concerning students’ mathematics performance, their score was based on an IRT model and not individual items, and it was therefore not possible to test for measurement invariance over time points. Third, following a person-centered approach (Bergman et al., 2003), students with similar patterns of academic well-being and mathematics performance were identified through latent profile analysis (LPA; Vermunt and Magidson, 2002). Next, configural frequency analyses (von Eye et al., 1996) were used to examine the stability of and changes in group memberships from Time 1 to Time 2. Finally, analyses of variance were conducted to examine how students within the different academic well-being and mathematics performance profiles differ with respect to their educational aspirations. We utilized the MPLUS (version 8) program (Muthén and Muthén, 1998–2015) to conduct the LCFAs and the LPAs. ANOVAs and chi-square tests were conducted with SPSS (Version 24).

#### Longitudinal Confirmatory Factor Analysis

The LCFA was performed simultaneously for seventh and ninth grade data on items representing academic well-being in Times 1 and 2. A model was specified in which all items for each scale were allowed to load on the corresponding factor only. On the basis of previous research, we expected that the items from the School Burnout Inventory (SBI; Salmela-Aro et al., 2009a) would load on three separate factors: exhaustion, cynicism, and inadequacy (Salmela-Aro et al., 2009a; Tuominen-Soini et al., 2012), that the nine items from the Schoolwork Engagement Inventory (EDA; Salmela-Aro and Upadyaya, 2012) would load on a single engagement factor, and that the three items measuring mathematics self-concept from Marsh’s (1992) Self Description Questionnaire I (SDQ I) would load on a single mathematics self-concept factor (Arens and Hasselhorn, 2015). We therefore specified a model with three school burnout factors (exhaustion, cynicism, and inadequacy), a schoolwork engagement factor, and a mathematics self-concept factor as factor indicators.

Due to slight non-normality in the items measuring burnout, we used maximum likelihood with robust standard errors as estimators in the analyses. We used chi-square (*χ*^2^), the comparative fit index (CFI), the Tucker–Lewis Index (TLI) and the root mean square error of approximation (RMSEA) as model-fit indicators. The CFI and TLI vary along a 0-to-1 continuum, and values greater than 0.90 and 0.95 reflect acceptable and excellent fit to the data, respectively. RMSEA values of less than 0.05 and 0.08 reflect a close fit and a reasonable fit to the data (Marsh et al., 2004). When comparing nested models, it is suggested that support for the more parsimonious model requires a change in the CFI of less than 0.01 and in the RMSEA of less than 0.015 (Chen, 2007). A prerequisite for meaningful comparison is that the measures are invariant across time points, that is, they measure the same underlying construct(s). LCFA allows for testing measurement invariance by specifying a series of nested models, where the endpoints are the least restrictive model with no invariance constraints, and the most restrictive model constraints all parameters to be the same across time points (Bollen, 1989). As a baseline model for testing measurement invariance, we specified a model imposing no invariance constraints on the factor loadings and indicator intercepts, assuming the same factor structure (configural invariance) at both time points. This model fitted the data very well. Next, we fitted a model where the factor loadings were constrained to equality (factorial invariance) across the time points, and this did not worsen the model fit. Finally, we compared the factorial invariance model against a fully invariant model, with both factor loadings and indicator intercepts constrained to equality (scalar invariance). The fully invariant model fitted the data well and did not worsen the model fit, indicating that the levels of the underlying items are equal at both time points. Therefore, the prerequisite for meaningful measurement invariance was achieved. The fit indices for the models tested are presented in **Table 2**.

**Table 2:** Goodness of fit statistics for alternative models.

Model	*χ*^2^	df	CFI	TLI	RMSEA	ΔCFI	ΔRMSEA	*p*
Configural invariance	2229.767	753	0.945	0.937	0.043			0.001
Factorial invariance	2266.531	774	0.945	0.938	0.042	0.000	0.001	0.000
Scalar invariance	2342.657	795	0.943	0.938	0.042	0.002	0.000	0.000

*CFI, comparative fix index; TLI, Tucker-Lewis index; RMSEA, root mean square error of approximation*.

#### Latent Profile Analyses

We used a person-centered approach to investigate what patterns of academic well-being and mathematics performance students show, and how stable these patterns are during one school year. The goal in the person-centered approach is to group students into categories, containing students who are similar to each other (Muthén and Muthén, 2000). To identify the cluster memberships in longitudinal data, we used the I-States as Objects Analysis (ISOA) procedure (Bergman and El-Khouri, 1999, 2003; Bergman et al., 2003), as it is optimal for studying short-term development. The key assumption for the ISOA approach is that the same typical patterns occur at all time points, although the proportion of the sample that belongs to each typical pattern may vary across time, and that some individuals may change the typical pattern they belong to. ISOA is based on data with the same set of variables measured at all time points, and the I-state is defined as an individual’s pattern of variable values at a specific time point. Since the present study consists of two time points, each person is characterized by two I-states. The I-states can therefore be identified despite of the time dimension, and the classification can be used to describe individual development.

First, our longitudinal data were reorganized in a way that the data for each student for both measurement points was coded as a separate case. Second, a series of LPAs (Muthén and Muthén, 2000; Vermunt and Magidson, 2002) was carried out on the reorganized data to identify students with similar patterns of academic well-being and mathematics performance. LPA is a probabilistic or model-based variant of traditional cluster analysis (Vermunt and Magidson, 2002). Its goal is to identify the smallest number of latent classes (groups) that adequately describe the associations among the latent continuous variables. In the analyses, one class is added in each step until the model optimally fits the data. To choose the best fitting model, Bayesian information criterion (BIC) and Vuong–Lo–Mendell–Rubin (VLMR) likelihood ratio test were used as the statistical criteria. The model with a lower BIC value is considered to provide a better fit to the data, and a resulting *p*-value of less than 0.05 for VLMR suggests that the estimated model is preferable over the reduced model (Lo et al., 2001). Furthermore, the usefulness and interpretableness of the latent classes (e.g., the number of individuals in each class) were also considered to choose the best fitting model. The LPAs were performed separately for seventh and ninth grade students and, in the LPA models, covariances were allowed to vary across clusters. Finally, the data were reorganized in such a way that the data for each student at both measurement points were again handled as two successive measurements of the same individual.

#### Configural Frequency Analyses

The stability of and changes in academic well-being and mathematics performance profiles (i.e., group memberships) within a school year (from Time 1 to Time 2) were examined through configural frequency analysis (CFA; von Eye, 1990). CFA compares the observed to expected frequencies in a cross-tabulation and asks whether cell frequencies are larger or smaller than could be expected by a base model. The base model selected for frequency comparison was the first-order CFA, which assumes that all variables under study may show main effects and are independent of each other (von Eye, 1990, 1996). By means of CFA, we searched for typical and atypical patterns. A type is a pattern that is observed more frequently than expected by chance, and an antitype is a pattern that is observed less frequently than expected by chance. In the present study, we focused on finding specific classes that the students tend to stay in more frequently than would be expected by chance (i.e., individual stability) as well as whether there are changes between the classes that cannot be ascribed to chance fluctuations (i.e., individual change).

#### Analyses of Variance

One-way ANOVAs were conducted to examine differences in academic well-being and mathematics performance between the profiles. ANOVAs were also conducted to investigate how students in the different academic well-being and mathematics performance profiles differ with respect to their educational aspirations at Time 1 and Time 2.

#### Chi-Square Test of Independence

A chi-square test was performed to examine the association between the academic well-being and mathematics performance profiles and gender. Then, we examined the adjusted residuals in each cell to see in which specific profile gendered differences occurred. If the residual exceeded the critical value of 1.96 in a z-distribution for either boys or girls, they were overrepresented in the profile.

## Results

### Academic Well-Being and Mathematics Performance Profiles

**FIGURE 1:**
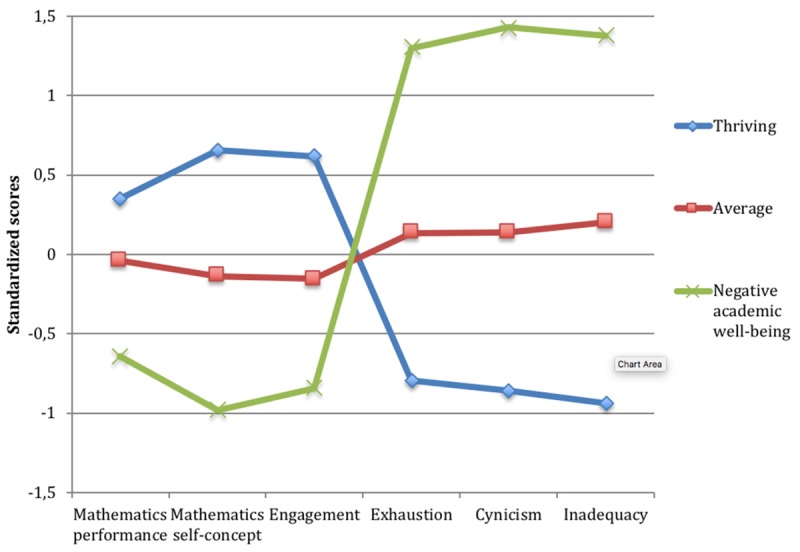
Seventh grade students’ latent mean scores on mathematics performance and academic well-being scales as a function of group membership.

**FIGURE 2:**
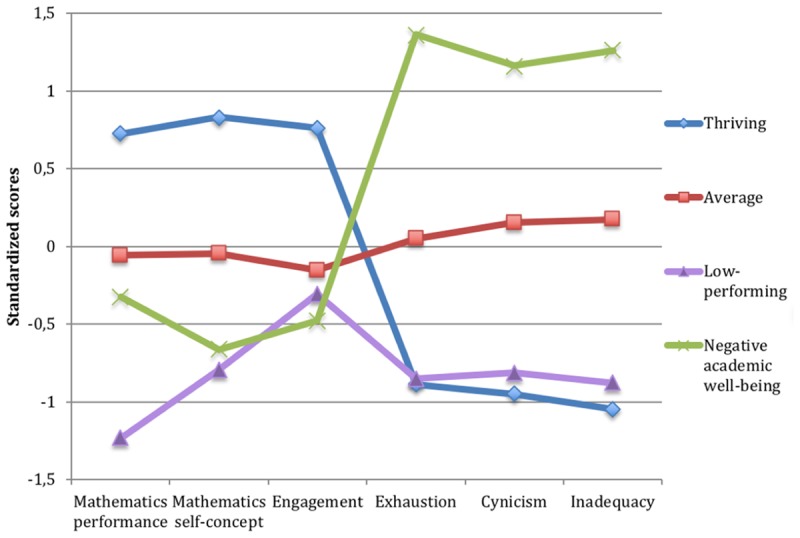
Ninth grade students’ latent mean scores on mathematics performance and academic well-being scales as a function of group membership.

The LPA results showed that a three-class solution described the data best for the seventh-grade students, whereas a four-class solution was supported among the ninth-grade students (see **Table 3** for fit indices). Although the BIC continued to decrease within both grades, the VLMR test clearly supported the three-class solution among the seventh-graders and the four-class solution among the ninth-graders. The entropy value was 0.79 for seventh grade and 0.80 for ninth grade, indicating that the models provide clear classifications. The average individual posterior probabilities for being assigned to a specific group are reported in **Appendix A**. The groups in each grade were labeled according to the mean score of their profiles in academic well-being and mathematics performance measures (grade 7 in **Figure 1** and grade 9 in **Figure 2**). The groups in seventh grade were named as thriving, average, and negative academic well-being. Since three of the groups in ninth grade were very similar to the three groups in seventh grade, the same names were used for both grades. The fourth group in ninth grade was named as low-performing students. H1 was thus confirmed in both grades, while H2 was supported in grade 9 but not in grade 7.

**Table 3:** Information criteria values for different class solutions in grade seven and nine.

Number of classes	Seventh grade			Ninth grade		
	BIC	*p*_V LMR_	Entropy	BIC	*p*_V LMR_	Entropy
1	19933.020	–	–	17019.537	–	–
2	18538.452	0.00	0.772	15961.446	0.00	0.737
3	18075.006	0.00	0.792	15557.889	0.00	0.789
4	17963.812	0.25	0.783	15454.759	0.00	0.803
5	17864.179	0.56	0.774	15367.386	0.05	0.765
6	17762.961	0.12	0.792	15290.460	0.23	0.774
7	17710.971	0.20	0.792	15240.417	0.20	0.781

*BIC, Bayesian Information Criterion; *p*_*VLMR*_, Vuong-Lo-Mendell-Rubin likelihood ratio test*.

ANOVAs were conducted to investigate the group differences in the mean scores of all measures (see grade 7 results in **Table 4**, grade 9 results in **Table 5**). The thriving group had the most positive performance and well-being in grade seven (*N*_I-States_ = 394, 33.8%; *N*_T1_ = 206, 35.3%; *N*_T2_ = 188, 32.2%) and grade nine (*N*_I-States_ = 245, 24.6%; *N*_T1_ = 114, 22.9%; *N*_T2_ = 131, 26.4%). The thriving students scored significantly higher in mathematics performance than the other groups and also had higher scores on mathematics self-concept and schoolwork engagement as well as relatively low scores on all dimensions of school burnout.

**Table 4:** Mean differences in mathematics performance and academic well-being measures between the profiles in grade seven.

Variable	Sample mean		Thriving		Average		Negative academic well-being		*F*(2,1163)	*p*	η^2^
	*N* = 1166		*N* = 394		*N* = 599		*N* = 173				
	*M*	*SD*	*M*	*SD*	*M*	*SD*	*M*	*SD*			
Mathematics performance	101.2	14.2	106.3	12.8	100.8	13.0	91.5	15.9	73.489	^∗^	0.11
Mathematics self-concept	3.6	0.91	4.2	0.67	3.4	0.76	2.6	0.90	275.431	^∗^	0.32
Engagement	4.2	1.5	5.1	1.2	3.9	1.3	2.9	1.4	207.173	^∗^	0.26
Exhaustion	2.7	1.1	1.8	0.65	2.9	0.81	4.2	1.0	586.527	^∗^	0.50
Cynicism	2.6	1.2	1.5	0.57	2.8	0.76	4.3	0.96	893.386	^∗^	0.61
Inadequacy	2.8	1.2	1.7	0.64	3.1	0.75	4.5	0.81	1005.085	^∗^	0.63

*Academic well-being and mathematics performance profile means within a row sharing the same subscripts are not significantly different at the *p* < 0.05 level. *N* describes the I-States rather than the number of students *^∗^p* < 0.001*.

**Table 5:** Mean differences in mathematics performance and academic well-being measures between the profiles in grade nine.

Variable	Sample mean		Thriving		Average		Low-performing		Negative academic well-being		*F*(3,990)	*p*	η^2^
	*N* = 994		*N* = 245		*N* = 501		*N* = 70		*N* = 178				
	*M*	*SD*	*M*	*SD*	*M*	*SD*	*M*	*SD*	*M*	*SD*			
Mathematics performance	109.4	15.6	121.2	12.8	108.3	13.1	88.9	14.6	104.2	13.6	131.068	^∗^	0.28
Mathematic self-concept	3.5	2.8	4.3	0.62	3.4	0.77	2.6_a_	0.80	2.9_a_	0.91	160.142	^∗^	0.33
Engagement	4.0	1.4	5.1	1.2	3.8_b_	1.2	3.6_ab_	1.5	3.3_a_	1.4	90.724	^∗^	0.22
Exhaustion	2.8	1.2	1.8_a_	0.67	2.9	0.72	1.7_a_	0.54	4.5	0.83	529.721	^∗^	0.62
Cynicism	2.6	1.2	1.6_a_	0.59	2.8	0.79	1.6_a_	0.55	4.0	0.97	410.244	^∗^	0.55
Inadequacy	3.0	1.3	1.7_a_	0.60	3.3	0.79	1.9_a_	0.69	4.7	0.71	654.868	^∗^	0.66

*Academic well-being and mathematics performance profile means within a row sharing the same subscripts are not significantly different at the *p* < 0.05 level. *N* describes the I-States rather than the number of students *^∗^ p* < 0.001*.

Students in the largest group, the average students (seventh grade: *N*_I-States_ = 599, 51.4%; *N*_T1_ = 300, 51.5%; *N*_T2_ = 299, 51.3%, ninth grade: *N*_I-States_ = 501, 50.4%; *N*_T1_ = 245, 49.3%; *N*_T2_ = 256, 51.5%) showed average scores on all measures, both in mathematics performance and in the academic well-being measures. Although the students in the average group had average scores on the measures, in comparison with the other groups, they still had the second highest score on both performance and burnout.

Students in the negative academic well-being group (seventh grade: *N*_I-States_ = 173, 14.8%; *N*_T1_ = 77, 13.2%; *N*_T2_ = 96, 16.5%, ninth grade: *N*_I-States_ = 178, 17.9%; *N*_T1_ = 97, 19.5%; *N*_T2_ = 81, 16.3%) expressed rather negative scores on all academic well-being measures. In both grades, students in the negative academic well-being group had significantly higher scores on all dimensions of school burnout in comparison with the other groups. The negative academic well-being group in seventh grade had the lowest scores in mathematics self-concept and engagement as well, while the ninth graders’ self-concept and engagement did not differ significantly from the low-performing students’ scores. Both the seventh and the ninth grade students in this group performed significantly lower in mathematics than the average students, however, there was a larger difference between the average and the negative academic well-being group in seventh grade (*d* = 0.6) than in ninth grade (*d* = 0.3). Further, the ninth-graders in the negative academic well-being group still performed significantly higher in mathematics than the low-performing students.

Finally, the fourth group found in ninth grade was the low-performing students (*N*_I-States_ = 70, 7.0%; *N*_T1_ = 41, 8.2%; *N*_T2_ = 29, 5.8%). These students performed the lowest in mathematics and had low scores on mathematics self-concept as well. However, interestingly, their scores on all dimensions of school burnout were quite positive and did not differ significantly from the scores of the thriving students. Also, their engagement in school was rather average.

### Gender Differences Within Academic Well-Being and Mathematics Performance Profiles

We examined the gender differences within the profiles by using a Chi-Square test, and found an association between gender and the academic well-being and mathematics performance groups in both seventh [χ^2^(2) = 35.119, *p* = 0.000] and ninth grade [χ^2^(3) = 50.223, *p* = 0.000]. A closer look at the adjusted residuals revealed that in both grades, there were significantly more girls in the negative academic well-being group (seventh grade: *N*_I-States boys_ = 56, 32%, *N*_I-States girls_ = 117, 68%, *z* = 5.0, *p* < 0.001, ninth grade: *N*_I-States boys_ = 47, 26%, *N*_I-States girls_ = 137, 74%, *z* = 6.2, *p* < 0.001), thus supporting H3. H4 was also confirmed, as boys were overrepresented in the thriving group (seventh grade: *N*_I-States boys_ = 233, 59%, *N*_I-States girls_ = 161, 41%, *z* = 4.6, *p* < 0.001, ninth grade: *N*_I-States boys_ = 140, 57%, *N*_I-States girls_ = 105, 43%, *z* = 3.5, *p* < 0.001). In ninth grade, there were more boys than girls in the low-performing group as well (*N*_I-States boys_ = 46, 66%, *N*_I-States girls_ = 24, 34%, *z* = 3.2, *p* = 0.002). In the remaining profiles, girls and boys were equally distributed.

### Change in and Stability of Academic Well-Being and Mathematics Performance Profiles

**FIGURE 3:**
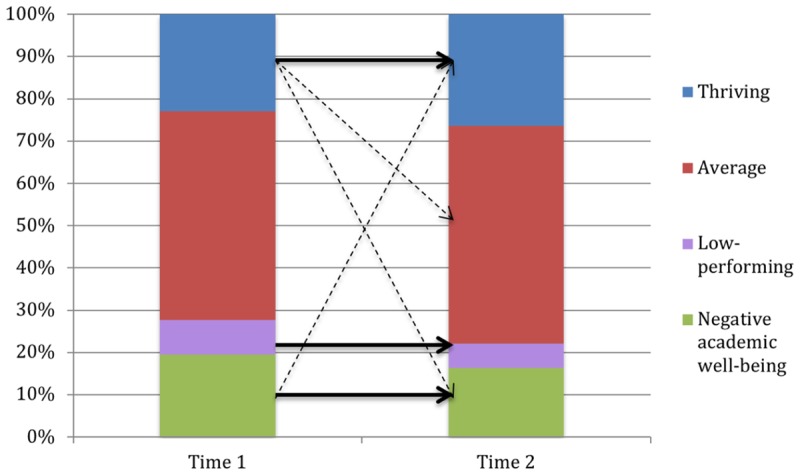
Statistical types and antitypes in seventh grade. Straight lines indicate pathways between time points identified as statistical types; broken lines indicate pathways identified as statistical antitypes.

**FIGURE 4:**
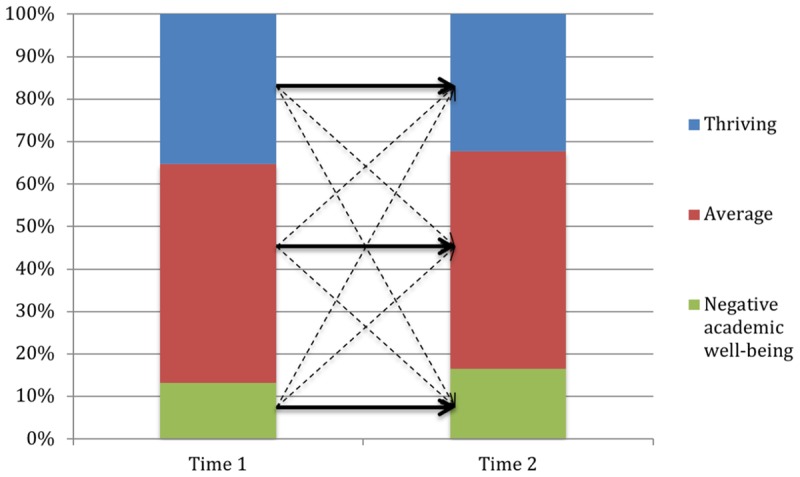
Statistical types and antitypes in ninth grade. Straight lines indicate pathways between time points identified as statistical types; broken lines indicate pathways identified as statistical antitypes.

Academic well-being and mathematics performance groups at Times 1 and 2 provided 9 configurations in seventh grade and 16 configurations in ninth grade. For each configuration, the observed frequency value was compared to the corresponding expected frequency value. To counter the inflation of Type-I error due to repeated significance testing, the Bonferroni-correction (0.05/number of tests) was applied to determine the significance of each configuration, resulting in a significance level of 0.006 in seventh grade and 0.003 in ninth grade. The CFA outcome for students in seventh grade is presented in **Table 6** and **Figure 3**, and for ninth grade in **Table 7** and **Figure 4**. In seventh grade, the CFA revealed three types and six antitypes. The three types that were revealed in the CFA corresponded to individuals belonging to the same class across both measurement points, thus supporting H5. Approximately 64% of the students in seventh grade displayed a stable academic well-being and mathematics performance profile over time. All remaining configurations represented antitypes, meaning that it was untypical for students in seventh grade to change between profiles during one school year. H6 and H7 were therefore supported as well.

**Table 6:** Configural frequency analysis on Time 1 and Time 2 academic well-being and mathematics performance groups in grade seven.

Configuration					
	T1/T2	OBS.	EXP.	*z*	*p*
T	1 1	129	66.43	8.16	0.00001
A	1 2	73	105.65	-3.51	0.00001
A	1 3	4	33.92	-5.29	0.00001
A	2 1	59	96.74	-4.20	0.00001
T	2 2	197	153.86	4.05	0.00001
A	2 3	44	49.40	-0.80	0.00001
A	3 1	0	24.83	-5.09	0.00001
A	3 2	29	39.49	-1.73	0.00001
T	3 3	48	12.68	10.03	0.00001

*T1, Time 1 academic well-being and mathematics performance group (1 = thriving, 2 = average, 3 = negative academic well-being). T2, Time 2 academic well-being and mathematics performance group (1 = thriving, 2 = average, 3 = negative academic well-being). A, Antitype; T, Type*.

**Table 7:** Configural frequency analysis on Time 1 and Time 2 academic well-being and mathematics performance groups in grade nine.

Configuration				
	T1/T2	OBS.	EXP.	*z*	*p*
T	1 1	75	30.05	8.46	0.00001
A	1 2	36	58.72	-3.16	0.001594
	1 3	2	6.65	-1.82	0.069524
A	1 4	1	18.58	-4.16	0.000032
	2 1	48	64.58	-2.21	0.027036
	2 2	151	126.20	2.56	0.010588
	2 3	10	14.30	-1.15	0.24891
	2 4	36	39.93	-0.65	0.516985
	3 1	3	10.81	-2.40	0.01635
	3 2	17	21.12	-0.92	0.359667
T	3 3	16	2.39	8.82	0.00001
	3 4	5	6.68	-0.66	0.512468
A	4 1	5	25.57	-4.18	0.00003
	4 2	52	49.96	0.30	0.761356
	4 3	1	5.66	-1.97	0.048838
T	4 4	39	15.81	5.93	0.00001

*T1, Time 1 academic well-being and mathematics performance group (1 thriving, 2 = average, 3 = low-performing 4 = negative academic well-being). T2, Time 2 academic well-being and mathematics performance group (1 thriving, 2 = average, 3 = low-performing 4 = negative academic well-being). A, Antitype; T, Type*.

In ninth grade, three types and three antitypes were found. Of four cells corresponding to individuals belonging to the same class across both time points, only three were significant types and H5 was therefore only partially confirmed. The one stable configuration that did not show to be a significant type, although it was close to reaching significance (*p* = 0.01), was the average group. Overall, approximately 57% of the students in ninth grade displayed a stable academic well-being and mathematics performance profile over time. In ninth grade, thriving students were unlikely to move to the average and the negative academic well-being group, and it was also untypical for students in the negative academic well-being group to move to the thriving group, thus, only supporting H7.

### Profile Differences in Educational Aspirations

Two One-way ANOVAs were performed to investigate how students with different profiles differ with respect to their educational aspirations. All effects and the mean differences between academic well-being and mathematics performance profiles in seventh grade are summarized in **Table 8**, and ninth grade in **Table 9**. One-way ANOVAs revealed significant differences in educational aspirations between the groups within both grades. The pairwise comparison of means in seventh grade revealed that thriving students had the highest aspirations compared to the other groups at both time points. The students in the average and negative academic well-being groups did not differ with respect to educational aspirations.

**Table 8:** Summary statistics and mean differences in educational aspirations between academic well-being and mathematics performance profiles in grade seven.

Variable	Thriving		Average		Negative academic well-being		*F*(2,580)	*p*	η^2^
	*M*	*SD*	*M*	*SD*	*M*	*SD*			
Educational aspirations T1	3.37	0.77	2.91_a_	0.81	2.80_a_	0.82	25.140	0.000	0.08
Thriving			*d* = 0.46		*d* = 0.57				
Average					*d* = 0.11				
Educational aspirations T2	3.33	0.80	3.04_a_	0.76	2.90_a_	0.83	11.859	0.000	0.04
Thriving			*d* = 0.29		*d* = 0.43				
Average					*d* = 0.14				

*Means within a row sharing the same letters are not significantly different at the *p* < 0.05 level (with Bonferroni correction). T1, Time 1; T2, Time 2*.

**Table 9:** Summary statistics and mean differences in educational aspirations between academic well-being and mathematics performance profiles in grade nine.

Variable	Thriving		Average		Low-performing		Negative academic well-being		*F*(3,493)	*p*	η^2^
	*M*	*SD*	*M*	*SD*	*M*	*SD*	*M*	*SD*			
Educational aspirations T1	3.54	0.65	3.13_a_	0.75	2.87_b_	0.84	3.13_ab_	0.79	11.790	0.000	0.07
Thriving			*d* = 0.41		*d* = 0.67		*d* = 0.41				
Average					*d* = 0.26		*d* = 0.00				
Low-performing							*d* = 0.26				
Educational aspirations T2	3.63	0.53	3.19_a_	0.68	2.60	0.84	3.11_a_	0.78	25.131	0.000	0.13
Thriving			*d* = 0.44		*d* = 1.03		*d* = 0.52				
Average					*d* = 0.59		*d* = 0.08				
Low-performing							*d* = 0.51				

*Means within a row sharing the same letters are not significantly different at the *p* < 0.05 level (with Bonferroni correction). T1, Time 1; T2, Time 2*.

The results for ninth grade students also showed that the thriving students had the highest educational aspirations in comparison with the other groups. Further, no significant differences were found between the average and the negative academic well-being students in neither of the time points. Lastly, the low-performing students had the lowest educational aspirations at Time 2, but interestingly, they did not differ in educational aspirations from the negative academic well-being students at Time 1. H8 was therefore confirmed in both seventh and ninth grade, while H9 was only partly confirmed in ninth grade, thus only at the second time point and only concerning the low-performing group but not the negative academic well-being group.

## Discussion

The aim of this study was to examine what kinds of profiles of academic well-being and mathematics performance can be found among students in seventh and ninth grade, if there are gender differences within the profiles, how stable the profiles are during one school year, and further, how students with different profiles differ with respect to their educational aspirations. Consistent with previous findings (Korhonen et al., 2014), in grade 9, we identified four distinct groups of students (thriving, average, negative academic well-being, and low-performing) that differed from each other in performance and well-being measures. Interestingly, we only found three groups of students in grade 7 (thriving, average, and negative academic well-being). Boys were overrepresented in the thriving group, while girls were overrepresented in the negative academic well-being group in both grades. The profiles were relatively stable during the school year, and it was highly unlikely, for example, for students to move between the thriving and negative academic well-being groups. Finally, in line with previous findings, students belonging to the thriving group held higher educational aspirations compared to the other groups. Contrary to our expectations, students belonging to the negative academic well-being group did not exhibit lower educational aspirations compared to the average group.

Regarding our first research question, we expected to find three groups of students based on the assumption that there is a linear relationship between academic well-being and performance (H1) but, also, that students with some sort of mixed profile, showing non-linear relations between well-being measures and performance, would emerge (H2). Three of these expected profiles were found within both seventh and ninth grade: a thriving, an average, and a negative academic well-being group. These profiles largely concur with the profiles found by Korhonen et al. (2014), even despite the use of slightly different indicators for academic well-being. A vast majority (approximately 80%) of students belonged to the thriving and the average profiles, displaying relatively positive mathematics performance and academic well-being. It is, however, notable that although the general assumption is that engagement and burnout are negatively associated (Schaufeli et al., 2002; Salmela-Aro et al., 2009b; Cadime et al., 2016), the average students seemed, as hypothesized based on previous results from studies using a person-centered approach (Tuominen-Soini and Salmela-Aro, 2014), to exhibit simultaneously average levels of both engagement and burnout.

The students in the third group identified in both grades, the negative academic well-being group, performed significantly lower in mathematics than the average students. However, the gap between the average and the negative academic well-being students’ mathematics performance was larger in seventh grade, while also, the negative academic well-being students in ninth grade performed significantly higher in mathematics than the low-performing group. In fact, the link between performance and well-being among the negative academic well-being students in seventh grade, resembles our assumptions made in our first hypothesis (H1), and is consistent with previous, linear findings suggesting that low performance is connected to higher levels of burnout (Salmela-Aro et al., 2009a), disengagement in school (Salmela-Aro and Upadyaya, 2014), and low academic self-concept (Valentine et al., 2004; Guo et al., 2015b). In turn, interestingly, the negative academic well-being profile in ninth grade has also some similar characteristics as the one we described in our second hypothesis (H2), as their mathematics performance is only slightly lower than the average students’. This association between performance and well-being has been found also in previous studies implementing a person-centered approach (Korhonen et al., 2014), and supports our assumption that there are non-linear relations between well-being and performance as well. As only three profiles were identified in seventh grade, it seems as if the negative academic well-being group in seventh grade represented students with both negative academic well-being and low performance, whereas these students were more separated in ninth grade (negative academic well-being, and low-performing). A plausible explanation for these findings is that negative well-being and low performance are not as clearly separated in the earlier years of adolescence, as they are later in ninth grade.

The profile representing low-performing students, only found in grade 9, showed somewhat ambiguous results with respect to the academic well-being measures. Regarding self-concept, the results are in line with previous research (Valentine et al., 2004; Guo et al., 2015b) and our hypothesis (H1), indicating that students with low performance report low academic self-concept as well. Indeed, these students performed the lowest in mathematics and their low performance was coupled with rather low mathematics self-concept. However, interestingly, the low-performing students still exhibited rather positive academic well-being. In fact, they did not differ significantly from the thriving students in school burnout nor did they differ from the average students in engagement. Overall, it seems like the students’ low mathematics performance and self-concept does not make them feel stressed out over, or overwhelmed by, schoolwork in general. It might be that the students in the low-performing group do not attach so much value to mathematics performance and, therefore, the domain-general constructs, engagement and school burnout, might not be as affected by the students’ low mathematics performance. This pattern resembles the one Tuominen-Soini et al. (2008) identified, in which students, despite their rather low academic achievement and motivation, still displayed less general distress and stress with their future aspirations than their more committed peers. Parhiala et al. (2018) also identified a similar group of students that, despite their low motivation, still had rather positive well-being. Although it is suggested that positive performance, motivation and well-being often go together, these results indicate that this is not always the case. It might be that some students are more psychologically detached from school than others, and that their well-being is more affected by experiences outside of school.

Next, with respect to our second research question, we investigated the distribution of gender within the profiles, and as hypothesized (H3), we found that girls were overrepresented in the negative academic well-being group, while there were more boys than expected among the thriving students (H4), within both seventh and ninth grade. In addition, boys were overrepresented in the low-performing group in ninth grade. Taken that girls have consistently been found to report higher levels of burnout than boys (Salmela-Aro et al., 2008; Salmela-Aro and Tynkkynen, 2012), our findings concur with these previous results, as the students in the negative academic well-being group indeed were characterized by high levels of exhaustion, cynicism, and inadequacy. This might also explain why boys were overrepresented in the groups displaying the lowest levels of burnout. Another explanation for the gendered differences within the profiles, could be that we only investigated well-being in relation to one domain, that is, mathematics. Boys have consistently been found to perform slightly better in mathematics than girls (Nagy et al., 2006; Watt et al., 2012), possibly due to gender stereotypes in mathematics, as they have been found to have a direct and indirect negative effect on girls’ mathematics performance (Song et al., 2016).

Regarding our third research question, we investigated the change in and stability of academic well-being and mathematics performance profiles. As all stable configurations in seventh grade, and three out of four stable configurations in ninth grade turned out to be significant types, H5 was, for the most part, supported. Further, changes between the thriving and the negative academic well-being groups were, as we expected (H7), untypical in both grades, whereas the assumption that changes between high-performing and low-performing students would be untypical as well (H6), was only supported among the seventh grade students. These findings resemble the results of previous investigations regarding the stability of and change in patterns of school burnout and engagement (Tuominen-Soini and Salmela-Aro, 2014) as well as patterns of perceived competence, academic value, and mental health (Roeser and Peck, 2003), suggesting continuity in adolescents’ academic and emotional functioning. In total, approximately 60% of the students displayed a stable academic well-being and mathematics performance profile over the course of one academic year and, although the remaining proportion of students in both grades showed a change in their academic well-being and mathematics performance profile, the majority of the changes that did occur were directed toward groups with fairly similar profiles. Also, only less than 10% of the students in each grade showed a considerably negative change (i.e., from the thriving or average group to the negative academic well-being or the low-performing group).

As for our final research question, regarding educational aspirations, our expectations came true regarding the thriving students in both seventh and ninth grade (H8), as they aspired for higher educational goals than students in the other groups. Previously, researchers have found that students with similar patterns of high engagement and low burnout not only have higher achievement (Fiorilli et al., 2017) and educational aspirations, but also, complete higher education studies (Tuominen-Soini and Salmela-Aro, 2014). Regarding the remaining groups, contrary to what we hypothesized (H9), no differences in educational aspirations were found between the negative academic well-being and the average students. One possible explanation is that students from both of these groups hold relatively high educational aspirations compared to their competence level and therefore have to invest more effort in schoolwork and consequently experience higher levels of school burnout (Tuominen-Soini and Salmela-Aro, 2014; Korhonen et al., 2016). In addition, the low-performing students only showed significantly lower aspirations than the other groups at the second time point. It might be that the students’ low mathematics performance and self-concept are affecting their educational aspirations negatively when they become faced with the decision of choosing an upper secondary education, possibly as they realize the demands of their initial aspirations, for example, the amount of schooling required or the enrollment criterions (Parker et al., 2013). Similar declines in educational aspirations over time has been found previously as well (Gottfredson, 1981, 1996).

As for practical implications, approximately 15% in seventh grade and 18% in ninth grade belonged to the negative academic well-being group. Since school burnout has been shown to have maladaptive consequences (Korhonen et al., 2014) and might even lead to later depressive symptoms (Bakker et al., 2000; Salmela-Aro et al., 2009b) it would be important to detect signs of poor academic well-being in an early stage. Also, because these students, despite their poor well-being, still have rather average performance, their potential problems might easily go unnoticed. Therefore, and since supporting students’ well-being plays a key role in the prevention of later health problems (van Uden et al., 2014), schools should screen students for well-being as well. There is also a need to provide and develop adequate coping skills for students and advise them on how to prevent and address their exhaustion and feelings of inadequacy as early as possible. Another interesting finding was that, although the negative academic well-being group in ninth grade performed significantly better in mathematics than the low-performing students, these groups shared the same level of low mathematics self-concept. It seems like the students in the negative academic well-being group might rate their performance lower than it actually is, therefore, it would be important to put more focus on improving students’ self-concept, for example, through praise and feedback, as these have been identified as very effective in enhancing self-concept (Craven et al., 2003; O’Mara et al., 2006). Also, since there is a strong, positive connection between academic self-concept and performance, findings indicate that interventions targeting both skills and self-concept within an academic domain (e.g., math) simultaneously are more effective (O’Mara et al., 2006).

Previous investigations have concluded that student’s engagement in schoolwork is significantly affected by teachers’ support in their efforts, as they play an important role in shaping students’ engagement through emotional, instructional, and organizational support embedded in the classroom processes (Anderson et al., 2004; van Uden et al., 2014). Our results illustrate that not only academic performance but, also, positive self-beliefs, engagement toward school and lack of school burnout matter for students’ educational aspirations. This should be recognized in an early stage, in order to help students set up challenging educational goals. This is also important, as we found that low-performing students’ educational aspirations were significantly lower than the other students’, just before the transition, at the time they were making the decision about their educational track in upper secondary education. As the last year of comprehensive school has been found to be a demanding phase for students (Salmela-Aro and Upadyaya, 2014), more attention and support should be given when the students are facing these important educational choices. Resources should be focused on, for example, the availability of school counselors and implementations of mental health-promotion programs in school, as they have been found to make positive changes in student’s social- and emotional skills, attitudes, and academic performance (e.g., Durlak et al., 2011; Sklad et al., 2012).

### Limitations and Future Research

Our study has several limitations that need to be taken into account. The three-class solution did not differentiate between students with low-performance and negative academic well-being. This might indicate that the students’ performance and well-being are not as clearly separated in the early years of adolescence. Future studies should investigate the relation between well-being and performance in younger students. Also, future research should incorporate different variables when investigating the relation between performance and well-being, for example, domain-specific task values, as they have revealed to be important predictors of students’ educational aspirations (Wigfield and Eccles, 2000). Of course, the applicability of the typology obtained in this study with Finnish adolescents should be tested in other cultures as well.

Further, since the measurement period in the present study was one year, it would be useful for future studies to investigate the development of academic well-being and performance over a longer time period. It would, for example, be important to follow the students to further education and working life, and investigate whether students with different patterns of well-being and performance differ later in their actual educational attainment level and career choices as well. Also, as we found that groups of students who experience both high and moderate levels of exhaustion, also hold relatively high educational goals compared to their competence level, it would be important to gain further insight into the sustained effects of academic well-being on young people’s educational aspirations and attainment. Future studies should, for example, investigate the reciprocal relationships between performance, well-being and educational aspirations over time.

## Conclusion

In conclusion, the present study contributes to the research on students’ academic well-being and performance in several ways. First, we demonstrated the added value of employing a person-centered approach when investigating the relation between academic well-being and mathematics performance among adolescent students, as we found both linear and non-linear relations between student’s academic well-being and performance. However, as only three profiles were identified in seventh grade, our results indicate that students’ well-being and performance are not as clearly separated in the earlier years of adolescence. Further, although our results show that the majority of students seem to perform quite well in mathematics and express a rather positive pattern of academic well-being and hold relatively high educational aspirations, it should be noted, that the proportion of students belonging to the negative academic well-being group was more than 15%. This, clearly, is something that should not be ignored as it has been suggested that school burnout may predict subsequent depressive symptoms later on (Salmela-Aro et al., 2009b). The present study also added to the existing research by investigating the stability of and change in academic well-being and mathematics performance profiles over one school year, and found that students’ academic well-being profiles are relatively stable. In our view, taking into account students’ mathematical skills as well as various aspects of academic well-being simultaneously, enables a more comprehensive understanding of students’ academic and emotional functioning and of the way it is linked with educational aspirations during critical transition periods, when adolescents are making choices regarding their future education and occupation. With this understanding, we might be able to identify the at-risk students and even to discover ways how to best support students to find suitable educational pathways for themselves and to thrive in school.

## Ethics Statement

This study was carried out in accordance with the recommendations of Finnish Advisory Board on Research Integrity guidelines, The Board for Research Ethics at Åbo Akademi University. The protocol was approved by The Board for Research Ethics.

## Author Contributions

Each author has made substantial contributions to the work. AW, HT, and JK designed this study. AW drafted the manuscript. HT and JK served as critical reviewers. AW and JK performed the research. AW analyzed the data. All authors approved the final version of the manuscript for submission.

## Conflict of Interest Statement

The authors declare that the research was conducted in the absence of any commercial or financial relationships that could be construed as a potential conflict of interest.

## Appendix

**Table A:** Average latent class probabilities for most likely latent class membership by latent class in seventh and ninth grade.

Most likely latent class membership	Latent class			
	(1) Thriving	(2) Average	(3) Low-performing	(4) Negative academic well-being
(1) Thriving	*0.904/0.899*	0.096/0.074	–/0.027	0.000/0.000
(2) Average	0.063/0.037	0.*900/0.896*	–/0.020	0.037/0.046
(3) Low-performing	-/0.078	-/0.124	*–/0.797*	–/0.000
(4) Negative academic well-being	0.000/0.000	0.088/0.103	–/0.000	*0.912/0.897*

*Values in italics represent the average posterior probability associated with the clusters to which students were assigned. Probabilities in seventh and ninth grade are separated by slash*.
